# Dual Anti-Metastatic and Anti-Proliferative Activity Assessment of Two Probiotics on HeLa and HT-29 Cell Lines 

**DOI:** 10.22074/cellj.2016.4307

**Published:** 2016-05-30

**Authors:** Zahra Nouri, Fatemeh Karami, Nadia Neyazi, Mohammad Hossein Modarressi, Roya Karimi, Mohammad Reza Khorramizadeh, Behrooz Taheri, Elahe Motevaseli

**Affiliations:** 1 Department of Medical Biotechnology, School of Advanced Technologies in Medicine, Tehran University of Medical Sciences, Tehran, Iran; 2 Department of Medical Genetics, School of Medicine, Tehran University of Medical Sciences, Tehran, Iran; 3 Department of Medical Biotechnology, International Campus, Tehran University of Medical Sciences, Tehran, Iran; 4 Department of Oncology, Kabul Medical University, Kabul, Afghanistan; 5 Department of Tissue Engineering, School of Advanced Technologies in Medicine, Tehran University of Medical Sciences, Tehran, Iran; 6 Department of Molecular Medicine, School of Advanced Technologies in Medicine, Tehran University of Medical Sciences, Tehran, Iran

**Keywords:** Probiotic, HT-29, HeLa, Metastasis, Gene Expression

## Abstract

**Objective:**

Lactobacilli are a group of probiotics with beneficial effects on prevention of cancer. However, there is scant data in relation with the impacts of probiotics in late-stage cancer progration, especially metastasis. The present original work was aimed to evaluate the anti-metastatic and anti-proliferative activity of *lactobacillus rhamnosus* supernatant (LRS) and *lactobacillus crispatus* supernatant (LCS) on the human cervical and colon adenocarcinoma cell lines (HeLa and HT-29, respectively).

**Materials and Methods:**

In this experimental study, the anti-proliferative activities of LRS and LCS were determined through MTT assay. MRC-5 was used as a normal cell line. Expression analysis of *CASP3, MMP2, MMP9, TIMP1* and TIMP2 genes was performed by quantitative reverse transcriptase-polymerase chain reaction (qRT-PCR), following the cell synchronization.

**Results:**

Supernatants of these two lactobacilli had cytotoxic effect on HeLa, however LRS treatment was only effective on HT-29 cell line. In addition, LRS had no side-effect on normal cells. It was shown that *CASP3* gene expression has been reduced after treatment with supernatants of two studied lactobacilli. According to our study, LRS and LCS are efficacious in the prevention of metastasis potency in HeLa cells with decreased expression of *MMP2, MMP9* and increased expression of their inhibitors. In the case of HT-29 cells, only LRS showed this effect.

**Conclusion:**

Herein, we have demonstrated two probiotics which have anti-metastatic effects on malignant cells and they can be administrated to postpone late-stage of cancer disease. LRS and LCS are effective on HeLa cell lines while only the effect of LRS is significant on HT-29, through cytotoxic and anti-metastatic mechanisms. Further assessments are required to evaluate our results on the other cancer cell lines, in advance to use these probiotics in other extensive trial studies.

## Introduction

Probiotics are nutritional supplements produced by viable non-pathogenic micro-organisms, inducing health benefits to the host (1). Probiotic bacteria have shown anti-tumor activities, leading to cancer risk reduction, by several mechanisms including production of anti-mutagenic compounds, degradation of carcinogenic compounds, changing concentration, function and metabolic features of micro-flora, host’s immune response alteration, and affecting the host physiology (2,3). They are effective in delaying cancer onset and progression as well as in controlling cell growth mechanisms (4,5). Most probiotics belong to the genus *Lactobacillus* which are part of the normal flora in healthy human vagina and have an important function in protecting the host from urogenital infections (6). The role of lactobacilli in modulation of systemic inflammation, apoptosis and cell proliferation as well as protection against pathogenic overgrowth has been demonstrated (7). Probiotic lactic acid bacteria (LAB) including L. acidophilus, L. casei, L. rhamnosus, B. longum, and B. lactis have been shown to decrease the incidence of carcinogen-induced colon tumors and precancerous lesions in experimental animals as well as in human (8,12). Although there is considerable evidence supporting the potential role of probiotic LAB in prevention of early stages colon cancer development, scant data exists pertaining to their role in later stages of colorectal cancer and cervical cancer, specifically in metastasis. Tissue invasion and metastasis is dependent on cell invasion through the extracellular matrix (ECM) and involves matrix metalloproteinases (MMPs) that degrade the ECM during the metastatic process (13,15). 

MMPs are zinc-dependent secreted proteinases which have critical role in promotion of tumor invasion through proteolytic induction of ECM components such as collagen, fibronectin, and gelatin (16). Recent studies also suggest that some MMPs play roles in tissue remodeling and wound healing (17,18). They are activated after secretion as pro-enzyme and cleaved extra-cellularly (19). TIMP-1 and TIMP-2 are two tissue inhibitors of MMP-9 and MMP-2, respectively (20). To evaluate whether lactobacilli treatment can affect late stages of cancer, specifically metastasis, in the human cervical cancer cells (HeLa) and human colon adenocarcinoma (HT-29), expression of the MMP-2, MMP-9, TIMP-1 and TIMP-2 genes were studied by quantitative reverse transcriptasepolymerase chain reaction (qRT-PCR). 

*Lactobacillus crispatus (L. crispatus)* and *Lactobacillus rhamnosus (L. rhamnosus)* are among the most abundant species in healthy women’s vagina (21). It has been shown that common vaginal lactobacilli are cytotoxic for cervical cancer cells, but not for normal cells, independently from pH and lactate (7). To assess the apoptotic effect of these lactobacilli on HeLa and HT-29 cells, gene expression of *CASP3* was also analyzed using qRT-PCR. 

## Materials and Methods

### Cell culture

In this experimental work, human cervical cancer cell line (HeLa), human colorectal adenocarcinoma cell line (HT-29) and human lung fibroblast (MRC-5) were purchased from Pasture Institute, National Cell Bank of Iran. The cells were cultured for 24 hours in Roswell Park Memorial Institute (RPMI) medium containing 10% fetal bovine serum (FBS), and 1% penicillin/streptomycin (all provided from Invitrogen, USA) in a humidified 37˚C atmosphere containing 5% CO_2_ . 

### Lactobacillus supernatant preparation

De Man Rogosa Sharpe (MRS) broth (pH=6.5, Merck, Germany) was used to grow L. crispatus strain SJ-3C-US and L. rhamnosus strain GG at 37˚C for 24 hours under micro-aerophilic conditions. Bacterial cultures (2×10^8^c.f.u./ml), which have been incubated for overnight, were centrifuged at 7000 rpm for 7 minutes. To remove remaining bacteria and debris the lactobacilli supernatants (LS) were filtered through a 0.2 mm membrane filter. In order to differentiate the effect of lactate produced by L. crispatus supernatant (LCS) and L. rhamnosus supernatant (LRS) with pH change impact, the pH in MRS (6.5) broth was adjusted to pH in LS (4.2 ± 0.1) with lactate, this control is called MRS with lactic acid (MRL). In this experiment, the following conditions were tested: LCS, pH=4.2; LRS, pH=4.2; MRS, pH=6.5; MRL, pH=4.2 in HeLa cells and LCS, pH=4.3; LRS, pH=4.3; MRS, pH=6.5; MRL, pH=4.3 in HT-29 cells. 

### MTT assay

MTT assay kit (Sigma, St. Louis, MO) was used to measure the inhibitory effect of LCS or LRS on HeLa, HT-29 and MRC-5 cell growth. A total of 10^4^ cells were seeded in each well containing RPMI medium, 10 % FBS, and 1% penicillin/streptomycin. After 24 hours, cells were treated with lactobacilli culture supernatants with concentration of 5, 10, 15, 20, 25% (v/v) in triplicates. Plates were incubated for 24 hours at 37˚C under 5% (v/v) CO_2_ . Cell viability was measured by ELISA reader (Anthons2020, version 1.8.3, UK) and analysis was performed using the following equation: 

Viability (percentage of the control)=[(absorbance of the sample-absorbance of the blank)/[absorbance of the control-absorbance of the blank)]×100 Cell synchronization for RNA extraction HeLa and HT-29 cells were seeded in RPMI medium containing 10% FBS, and 1% penicillin/ streptomycin for 24 hours. Subsequently, each cell line was counted and equal number of the cells were sub-cultured in four 25-cm ^3^flasks and synchronized, three of which were selected to be treated with LS, MRL and MRS for 4 hours. The last flask was used as control, without any treatment. 

### RNA isolation, cDNA synthesis and quantitative reverse transcriptase-polymerase chain reaction

Total RNA was isolated from each treated and non-treated cells using TriPure Reagent kit (Roshe Applied Science, Germany). RNA quality and quantity were determined using Nanodrop, spectrophotometrically (Thermo Scientific, USA). PrimeScript RT reagent kit (Takara Bio, Japan) was used for reverse transcription of RNA and then mRNA expression of target genes were analyzed using qRT-PCR. Real-time quantification of purified mRNA genes including *MMP2, MMP9, TIMP1, TIMP2, CASP3* and PGM were performed using rotor gene 6000 (Corbet, Australia). Real-time master mix reaction was comprised of 2x master mix (Takara Bio, Japan), 250 ng cDNA, 10 pmol of each primer pairs adjusted with ddH_2_O up to final reaction of 10 µl. Primer sequences for all of the studied genes were picked up from previous reports and were checked again using online Primer 3 software and NCBI-BLAST (22,27). List of primer sets are available in the Table 1. The thermal cycling program was consisted of an initial cDNA denaturation at 95˚C for 10 seconds, following 50 cycles of two steps amplification, including denaturation at 95˚C for 10 seconds and annealing as well as extension at 65˚C for 30 seconds. The experiments were performed in duplicate for each data point. PGM gene was selected as a normalizer. To ensure the specificity of qRT-PCR products and absence of primer dimer, melting curve analysis was performed after each run of amplification. 

**Table 1:** Sequence of the primers applied for qRT-PCR


Primer	Primer sequences	Product size (bp)	References

MMP2	F:GCAGTGCAATACCTGAACACC	111	22
	R: GTCTGGGGCAGTCCAAAGAACT		
MMP9	F:GCACGACGTCTTCCAGTACC	124	23
	R: CAGGATGTCATAGGTCACGTAGC		
TIMP1	F:TTCTGGCATCCTGTTGTTGCT	106	24
	R: CCTGATGACGAGGTCGGAATT		
TIMP2	F:TGGAAACGACATTTATGGCAACCC	146	25
	R: CTCCAACGTCCAGCGAGACC		
CASP3	F:ACATGGCGTGTCATAAAATACC	120	26
	R: CACAAAGCGACTGGATGAAC		
PGM	F:AGCATTCCGTATTTCCAGCAG	120	27
	R: GCCAGTTGGGGTCTCATACAAA		


qRT-PCR; Quantitative reverse transcriptase-polymerase chain reaction.

#### Statistical analysis

For statistical analysis of the intended genes, relative expression and group-wise comparison of their total expression between treated and control states, a randomization Excel-based test in relative expression software tool was used. Mann-Whitney test with SPSS software was used to calculate the half maximal inhibitory concentration (IC_50_). To do this, the treated cell IC_50_ was compared to MRL and control cells IC_50_ . Mean ± SE was used to express all data in three separate experiments and P<0.05 was considered as significant threshold. 

### Results

#### Cytotoxic effect of L. crispatus strain SJ-3C-US and L. rhamnosus strain GG culture supernatants on HeLa, HT-29 and MRC-5 cell growth 

**Fig.1:**
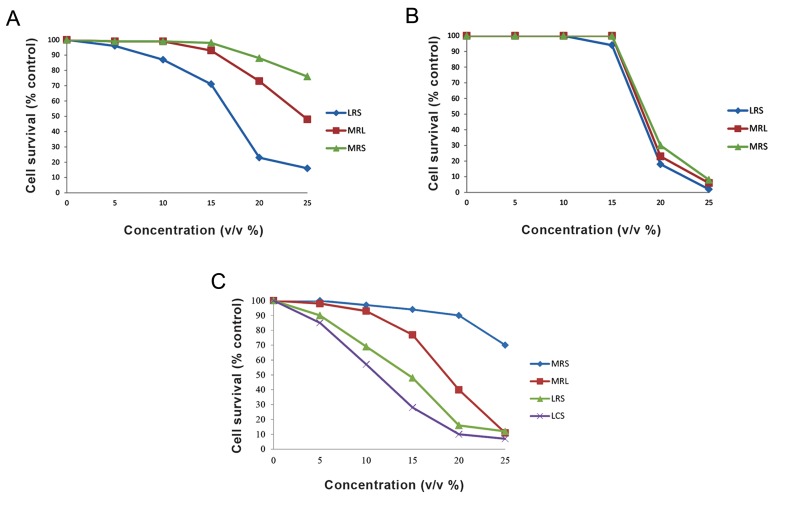
The images represent cytotoxicity effects of LRS, MRL and MRS with different concentrations on A. HT-29 cells, B. MRC-5 as normal cells, as well as LCS, LRS, MRL and MRS on C. HeLa cell line measured by MTT assay. The mean value is represented with three separate experiments for each point. LRS; *Lactobacillus rhamnosus* supernatant, MRS; De Man Rogosa Sharpe, MRL; MRS with lactic acid, and LCS; *Lactobacillus crispatus* supernatant.

Cell growth inhibitory effects were determined by MTT assay (Fig.1). In this experiment, MRC-5 was used as a normal cell line. Findings demonstrated that LCS and LRS had a significant inhibitory effect on HeLa cell growth in comparison with the cells treated with control MRL and MRS solutions. These results showed that the acidity was not the main cause of HeLa cell growth inhibition. In addition, the IC_50_ value of LCS and LRS against HeLa cells was respectively 11 and 15% (v/v), proposing that a substance, other than lactate, in the LRS and LCS could only affect the cervical tumor cells (HeLa) but not the normal cells. In contrast to LCS, significantly higher growth inhibitory effect was observed by treatment of the HT-29 cells with LRS. Further analyses showed 17% (v/v) IC_50_ value for HT-29 treated cells with LRS. Curiously, these findings also suggest the presence of a substance other than lactate in the LRS which can specifically target HT-29 cancer cells but not the normal cell (MRC-5). No sideeffect was detected on the MRC-5 normal cells treated with LRS. 

### *MMP2, MMP9, TIMP1, TIMP2* and *CASP3* genes expression in HeLa and HT-29 cells treated with LRS or LCS 

**Fig.2:**
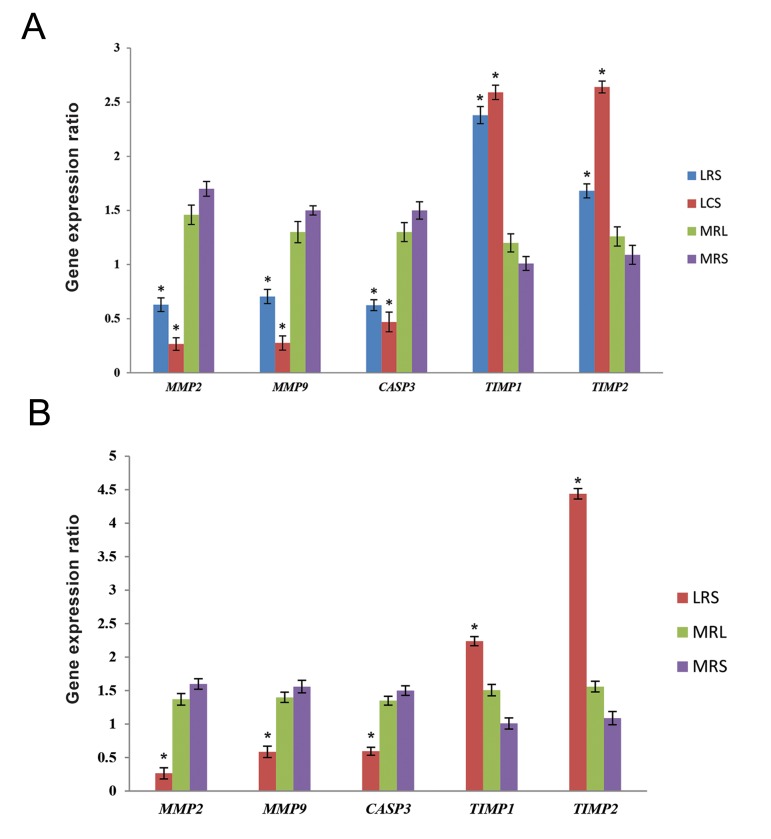
Images show the effect of lactobacilli supernatant on mRNA expression level of *MMP2, MMP9, TIMP1, TIMP2* and *CASP3* genes in the treated HeLa cells with LRS, LCS, MRS and MRL or treated HT-29 cells with LRS, MRS and MRL. *; P<0.05, LRS; *Lactobacillus rhamnosus* supernatant, MRS; De Man Rogosa Sharpe, MRL; MRS with lactic acid, and LCS; *Lactobacillus crispatus* supernatant.

mRNA expression level of *MMP2, MMP9, TIMP1, TIMP2* and *CASP3* genes were quantified by qRT-PCR after 4 hours treatment with LCS, LRS, MRL or MRS. We determined that mRNA level of *MMP2, MMP9* and *CASP3* genes were down-regulated, while *TIMP1* and *TIMP2* expression levels were up-regulated in the HeLa cells treated with LRS or LCS, compared to those cells treated with MRL or MRS. Similarly, LRS down-regulated the mRNA expression level of *MMP2, MMP9* and *CASP3* genes when *TIMP1* and *TIMP2* expression levels were upregulated, in comparison with MRL or MRS treated HT-29 cells; but LCS had no significant effect on the expression level of *MMP2, MMP9, TIMP1, TIMP2* and *CASP3* genes in HT-29 cells (Fig.2). 

### Discussion

It has been demonstrated that probiotics could play anti-cancer roles by contributing to several mechanisms, including induction of immune responses as well as anti-proliferative, anti-apoptotic or anti-microbial activities. Evidences revealed that primary colorectal and cervical cancer cells could reach to the other organs such as liver, lung and brain (28,29) by activating *MMPs* (e.g. *MMP2* and *MMP9*) and subsequently degradation of the ECM (30). 

We have previously demonstrated that lactobacilli culture supernatants caused significant cytotoxic effect on cervical cancer cells (7). In this study, we have sought the effects of L. crispatus or L. rhamnosuson culture supernatant treatment on HeLa, HT29 and MRC-5 cell growth, using MTT assay and transcriptional analysis of some metastatic genes including MMP-2, MMP-9 and relevant inhibitors. We determined down-regulation of *MMP-2* and *MMP-9* as well as up-regulation of *TIMP-1* and *TIMP-2* gene expression in HeLa and HT-29 cell lines by treatment with LRS, proposing the inhibitory effect of this probiotic on metastasis of cervical and colorectal cancers. Consistent with our findings, L. rhamnosus and L. casei were previously shown to have an inhibitory effect on MMP9 enzymatic activity, while they increased the expression of zona occludense-1, as a critical protein preventing of metastasis in HCT-116 cells (31). In the present study, both LRS and LCS indicated anti-proliferative effect on HeLa cell growth, while only LRS showed an effective role on prohibition of HT-29 cell proliferation. Herein, expression of *CASP3* gene (with critical roles in apoptosis) was down-regulated by lactobacilli culture supernatants, confirming, our previously reported results (7). Thus far, several studies indicated that probiotic lactic acid bacteria, including L. acidophilus, L. casei, L. rhamnosus, B. longum, and B. lactis supernatants, are able to inhibit colorectal cancer progressions (8,12). It has previously been demonstrated that supernatant of L. Delbrueckii could reduce SW620 colon cancer cell growth by inducing apoptosis through intrinsic CASP-3 dependent pathways. It has been shown that MMP9 enzymatic activity was decreased in the cells treated with supernatant of L. Delbrueckii (32). In addition, treatment of Caco-2 cell line with either L. acidophilus or L. Casei supernatants could increase apoptosis, consequently hindering the cancer cell migrations and invasions (33). Moreover, it was shown that viable or heat-killed L. paracasei IMPC2.1 and L. rhamnosus GG had anti-apoptotic and anti-proliferative effects on HGG and DLD-1 (34). Inducing autophagy pathway is the other proposed anti-proliferative mechanism of probiotics while as we demonstrated several genes with crucial autophagy roles were down-regulated in HeLa cells, due to treatment with LCS or LRS (35). However, cell-bound exo-polysaccharides of L. acidophilus 606 have an anti-tumor activity against malignancy in HT-29 cell line, by activating effective genes involved in autophagy pathway including Beclin-1 and GRP78 (36). Therefore, we can deduce that these two lactobacilli culture supernatants are not effective in autophagy and apoptotic process. To determine the pivotal keys contributed to HeLa cell death, further investigations on necrosis procedure are required. 

In the present study, we have demonstrated that treatment with LRS had no cytotoxic effect on MRC-5 cells, as a normal cell line. A recent study has investigated L. acidophilus 36YL strain metabolites secretion on different cancerous cell lines including HeLa, MCF-7, AGS and HT-29 compared to the normal cells (HUVEC). The metabolites of these bacteria decreased viability in all of the cancerous cell lines with no toxic effect on the normal cells (37). Herein, in comparison with MRL and LRS (at similar pH condition), no cytotoxicity effect was observed in the normal cell line treated with LRS. Regarding the fact that, one of the principle objectives in cancer therapies is limiting damage of normal cells and tissue, it could be further highlighted that LRS contains anti-tumor substances, inducing neither acidity nor cytotoxicity effect on normal cells, compared to MRL. 

## Conclusion

Probiotics include the major normal flora of colon and cervix. They can open a new way toward prevention or even suppression of cervical cancer and colorectal cancer cell invasions. Further investigations are required to focus on supernatant fraction and assess the effect of these fractions on different cancer cells. 

## References

[1] Aureli P, Capurso L, Castellazzi AM, Clerici M, Giovannini M, Morelli L (2011). Probiotics and health: an evidencebased review. Pharmacol Res.

[2] Rafter J (2003). Probiotics and colon cancer. Best Pract Res Clin Gastroenterol.

[3] Saikali J, Picard C, Freitas M, Holt P (2004). Fermented milks, probiotic cultures, and colon cancer. Nutr Cancer.

[4] Linsalata M, Russo F, Berloco P, Valentini AM, Caruso ML, De Simone C (2005). Effects of probiotic bacteria (VSL# 3) on the polyamine biosynthesis and cell proliferation of normal colonic mucosa of rats. In Vivo.

[5] de Moreno de Leblanc A, Perdigón G (2010). The application of probiotic fermented milks in cancer and intestinal inflammation. Proc Nutr Soc.

[6] McLean NW, Rosenstein IJ (2000). Characterisation and selection of a Lactobacillus species to re-colonise the vagina of women with recurrent bacterial vaginosis. J Med Microbiol.

[7] Motevaseli E, Shirzad M, Akrami SM, Mousavi AS, Mirsalehian A, Modarressi MH (2013). Normal and tumour cervical cells respond differently to vaginal lactobacilli, independent of pH and lactate. J Med Microbiol.

[8] McIntosh GH, Royle PJ, Playne MJ (1999). A probiotic strain of L.acidophilus reduces DMH-induced large intestinal tumors in male Sprague-Dawley rats. Nutr Cancer.

[9] Rowland IR, Bearne CA, Fischer R, Pool-Zobel BL (1996). The effect of lactulose on DNA damage induced by DMH in the colon of human flora-associated rats. Nutr Cancer.

[10] Burns AJ, Rowland IR (2004). Antigenotoxicity of probiotics and prebiotics on faecal water-induced DNA damage in human colon adenocarcinoma cells. Mutat Res.

[11] Rafter J, Bennett M, Caderni G, Clune Y, Hughes R, Karlsson PC (2007). Dietary synbiotics reduce cancer risk factors in polypectomized and colon cancer patients. Am J Clin Nutr.

[12] Yamazaki K, Tsunoda A, Sibusawa M, Tsunoda Y, Kusano M, Fukuchi K (2000). The effect of an oral administration of Lactobacillus casei strain shirota on azoxymethaneinduced colonic aberrant crypt foci and colon cancer in the rat. Oncol Rep.

[13] Leeman MF, Curran S, Murray GI (2003). New insights into the roles of matrix metalloproteinases in colorectal cancer development and progression. J Pathol.

[14] Mook OR, Frederiks WM, Van Noorden CJ (2004). The role of gelatinases in colorectal cancer progression and metastasis. Biochim Biophys Acta.

[15] Rahim F, Hajizamani S, Mortaz E, Ahmadzadeh A, Shahjahani M, Shahrabi S (2014). Molecular regulation of bone marrow metastasis in prostate and breast cancer. Bone Marrow Res.

[16] Pulkoski-Gross AE (2015). Historical perspective of matrix metalloproteases. Front Biosci (Schol Ed).

[17] Syggelos SA, Aletras AJ, Smirlaki I, Skandalis SS (2013). Extracellular matrix degradation and tissue remodeling in periprosthetic loosening and osteolysis: focus on matrix metalloproteinases, their endogenous tissue inhibitors, and the proteasome. Biomed Res Int.

[18] Hameedaldeen A, Liu J, Batres A, Graves GS, Graves DT (2014). FOXO1, TGF-β regulation and wound healing. Int J Mol Sci.

[19] Clark IM, Swingler TE, Sampieri CL, Edwards DR (2008). The regulation of matrix metalloproteinases and their inhibitors. Int J Biochem Cell Biol.

[20] Shi M, Cao M, Song J, Liu Q, Li H, Meng F (2015). PinX1 inhibits the invasion and metastasis of human breast cancer via suppressing NF-κB/MMP-9 signaling pathway. Mol Cancer.

[21] Motevaseli E, Shirzad M, Raoofian R, Hasheminasab SM, Hatami M, Dianatpour M (2013). Differences in vaginal lactobacilli composition of Iranian healthy and bacterial vaginosis infected women: a comparative analysis of their cytotoxic effects with commercial vaginal probiotics. Iran Red Crescent Med J.

[22] Cui HY, Guo T, Wang SJ, Zhao P, Dong ZS, Zhang Y, et al (2012). Dimerization is essential for HAb18G/CD147 promoting tumor invasion via MAPK pathway. Biochem Biophys Res Commun.

[23] Safranek J, Pesta M, Holubec L, Kulda V, Dreslerova J, Vrzalova J (2009). Expression of MMP-7, MMP-9, TIMP1 and TIMP-2 mRNA in lung tissue of patients with nonsmall cell lung cancer (NSCLC) and benign pulmonary disease. Anticancer Res.

[24] Shynlova O, Bortolini MA, Alarab M (2013). Genes responsible for vaginal extracellular matrix metabolism are modulated by women’s reproductive cycle and menopause. Int Braz J Urol.

[25] Menon A, Pettinari L, Martinelli C, Colombo G, Portinaro N, Dalle-Donne I (2013). New insights in extracellular matrix remodeling and collagen turnover related pathways in cultured human tenocytes after ciprofloxacin administration. Muscles Ligaments Tendons J.

[26] Xu M, Takanashi M, Oikawa K, Tanaka M, Nishi H, Isaka K (2009). USP15 plays an essential role for caspase-3 activation during Paclitaxel-induced apoptosis. Biochem Biophys Res Commun.

[27] Mobasheri MB, Shirkoohi R, Zendehdel K, Jahanzad I, Talebi S, Afsharpad M (2015). Transcriptome analysis of the cancer/testis genes, DAZ1, AURKC, and TEX101, in breast tumors and six breast cancer cell lines. Tumour Biol.

[28] LeGolvan MP, Resnick M (2010). Pathobiology of colorectal cancer hepatic metastases with an emphasis on prognostic factors. J Surg Oncol.

[29] Setoodeh R, Hakam A, Shan Y (2012). Cerebral metastasis of cervical cancer, report of two cases and review of the literature. Int J Clin Exp Pathol.

[30] Lu P, Takai K, Weaver VM, Werb Z (2011). Extracellular matrix degradation and remodeling in development and disease. Cold Spring Harb Perspect Biol.

[31] Escamilla J, Lane MA, Maitin V (2012). Cell-free supernatants from probiotic Lactobacillus casei and Lactobacillus rhamnosus GG decrease colon cancer cell invasion in vitro. Nutr Cancer.

[32] Wan Y, Xin Y, Zhang C, Wu D, Ding D, Tang L (2014). Fermentation supernatants of Lactobacillus delbrueckii inhibit growth of human colon cancer cells and induce apoptosis through a caspase 3-dependent pathway. Oncol Lett.

[33] Soltan Dallal MM, Mojarrad M, Baghbani F, Raoofian R, Mardaneh J, Salehipour Z (2015). Effects of probiotic Lactobacillus acidophilus and Lactobacillus casei on colorectal tumor cells activity (CaCo-2). Arch Iran Med.

[34] Orlando A, Refolo M, Messa C, Amati L, Lavermicocca P, Guerra V (2012). Antiproliferative and proapoptotic effects of viable or heat-killed Lactobacillus paracasei IMPC2.1 and Lactobacillus rhamnosus GG in HGC-27 gastric and DLD-1 colon cell lines. Nutr Cancer.

[35] Motevaseli E, Azam R, Akrami SM, Mazlomy M, Saffari M, Modarressi MH (2016). The effect of Lactobacillus crispatus and Lactobacillus rhamnosus culture supernatants on expression of autophagy genes and HPV E6 and E7 oncogenes in the HeLa cell line. Cell J.

[36] Kim Y, Oh S, Yun HS, Oh S, Kim SH (2010). Cell-bound exopolysaccharide from probiotic bacteria induces autophagic cell death of tumour cells. Lett Appl Microbiol.

[37] Nami Y, Abdullah N, Haghshenas B, Radiah D, Rosli R, Khosroushahi AY (2014). Probiotic potential and biotherapeutic effects of newly isolated vaginal Lactobacillus acidophilus 36YL strain on cancer cells. Anaerobe.

